# Brain midline shift assessment using sonography in neurocritical care patients

**DOI:** 10.1186/cc9763

**Published:** 2011-03-11

**Authors:** J Motuel, I Biette, C Cognard, O Fourcade, T Geeraerts

**Affiliations:** 1 University Hospital, Toulouse, France

## Introduction

Brain midline shift (MLS) is a life-threatening condition that requires urgent diagnosis and treatment [1]. Bedside MLS assessment with sonography has been proposed as a valuable method in stroke patients [2]. We aimed to validate this method in neurocritical care patients by comparing it with the brain CT gold standard method.

## Methods

This prospective study was conducted in a single neurocritical care unit. Patients who underwent brain CT scan were included and a concomitant brain sonography with MLS measurement was performed. Using sonography, the midline was determined bilaterally with a 2 to 4 MHz probe using the temporal window by visualizing the third ventricle, with a double hyperechogenic image above the mesencephalon. MLS was calculated as the difference between both sides for midline line measurements. CT MLS was independently calculated by a specialist in neuroradiology as the maximal difference between the ideal midline and the actual interventricular septum. A significant MLS was defined on brain CT as >0.5 cm.

## Results

**Figure 1:**
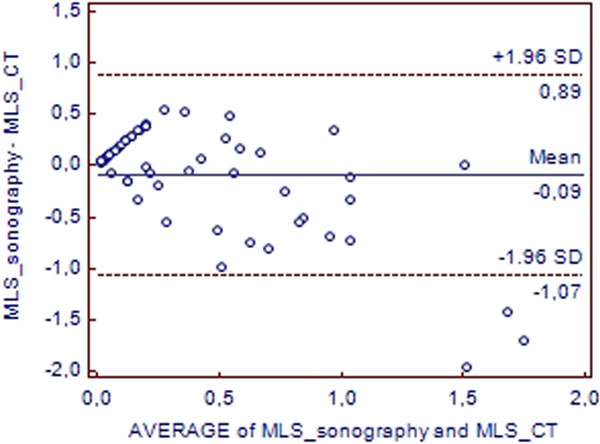
**Bland-Altman plot: agreement between sonography and CT for MLS assessment**.

Fifty-five patients (with a total of 67 paired measured) were included (72% male with a median IGS II of 35.5 ranging from 12 to 65) (35 TBI, eight subarachnoidal hemorrhage, five intracerebral hematoma, seven postoperative care). The mean (± SD) MLS was 0.34 ± 0.34 cm using sonography and 0.48 ± 0.68 cm using CT. The linear regression showed an *r *value at 0.64 between sonographic and CT MLS (*P *< 0.0001). Bland-Altman plot showed a mean bias of 0.09 cm and three values out of the limits of agreement (4% of the total measures) (Figure 1). For sonography, the area under ROC curve for the detection of significant MLS was 0.80 (0.68 to 0.89) with a best cut-off value of 0.46 cm with 74% sensitivity and 89% specificity.

## Conclusions

MLS measurement using sonography appears to have interesting performances for the detection of significant MLS (that is, >0.5 cm on brain CT). As the regression between sonographic and CT values for MLS was not very strong, and as the agreement between both methods showed relatively large limits of agreements, sonography would not replaced the gold standard CT method. However, the bedside estimate could be used as a detection tool in emergency in neurocritical care patients.
